# The aerodynamics of flight in an insect flight-mill

**DOI:** 10.1371/journal.pone.0186441

**Published:** 2017-11-01

**Authors:** Gal Ribak, Shay Barkan, Victoria Soroker

**Affiliations:** 1 School of Zoology, Faculty of Life Sciences, Tel Aviv University, Tel Aviv, Israel; 2 The Steinhardt Museum of Natural History and Israel National Center for Biodiversity Studies, Tel Aviv, Israel; 3 Department of Entomology, Agricultural Research Organization, Volcani Center, Rishon LeZion, Israel; Coastal Carolina University, UNITED STATES

## Abstract

Predicting the dispersal of pest insects is important for pest management schemes. Flight-mills provide a simple way to evaluate the flight potential of insects, but there are several complications in relating tethered-flight to natural flight. We used high-speed video to evaluate the effect of flight-mill design on flight of the red palm weevil (*Rynchophorous ferruginneus*) in four variants of a flight-mill. Two variants had the rotating radial arm pivoted on the main shaft of the rotation axis, allowing freedom to elevate the arm as the insect applied lift force. Two other variants had the pivot point fixed, restricting the radial arm to horizontal motion. Beetles were tethered with their lateral axis horizontal or rotated by 40°, as in a banked turn. Flight-mill type did not affect flight speed or wing-beat frequency, but did affect flapping kinematics. The wingtip internal to the circular trajectory was always moved faster relative to air, suggesting that the beetles were attempting to steer in the opposite direction to the curved trajectory forced by the flight-mill. However, banked beetles had lower flapping asymmetry, generated higher lift forces and lost more of their body mass per time and distance flown during prolonged flight compared to beetles flying level. The results indicate, that flapping asymmetry and low lift can be rectified by tethering the beetle in a banked orientation, but the flight still does not correspond directly to free-flight. This should be recognized and taken into account when designing flight-mills and interoperating their data.

## Introduction

Flight-mills are often used to study the potential of insects to make long migratory flights [1–8]. While flight-mills come in different shapes and sizes, their basic principle is the same. An insect is tethered to a radial horizontal beam which is free to rotate, at low friction, about a vertical shaft. As the insect flaps its wings to fly forward, it is restricted to flying in a horizontal circle with the perimeter dictated by the radial beam length. Miller [8] cites a flight-mill study from more than a century ago [9], but much of the current flight-mill research in insects is derived from the roundabouts of Kennedy et al. [10] and Krogh and Weis-Fogh [11]. In those studies, locusts were tethered to a large roundabout, which was rotated so that the oncoming air flow and perceived sense of motion would stimulate the insects to flap their wings and make prolonged tethered flights. The roundabout, thus provided a simple assay to replace tethered flight in a wind tunnel. In their seminal paper, Krogh and Weis-Fogh [11] described that, to improve Kennedy et al.’s roundabout, they suspended each tethered insect in such a way that it swung outwards under centrifugal force. The resultant of gravity and centrifugal force thus acted ventrally upon to the insects and in their sagittal plane. This diminished the outward bending of the body that was evident in Kennedy et al.’s [10] photographs.

In contrast to the roundabouts that were rotated by mechanical means, most current flight-mills are smaller and have very low torsional torque so that the tethered flight of the insect is sufficient to rotate the flight-mill; i.e., the rotation of the flight-mill is driven by the flapping wings and flight muscles and the insect can start and stop flight voluntarily and control its flight speed. Such flight-mills provide a very convenient, and often automated, means to measure the flight of insects in the laboratory [12]. However, it is not possible to accurately convert the distances flown in a flight-mill to distances flown in the wild [13–15], due to three main reasons.

**Fig 1:**
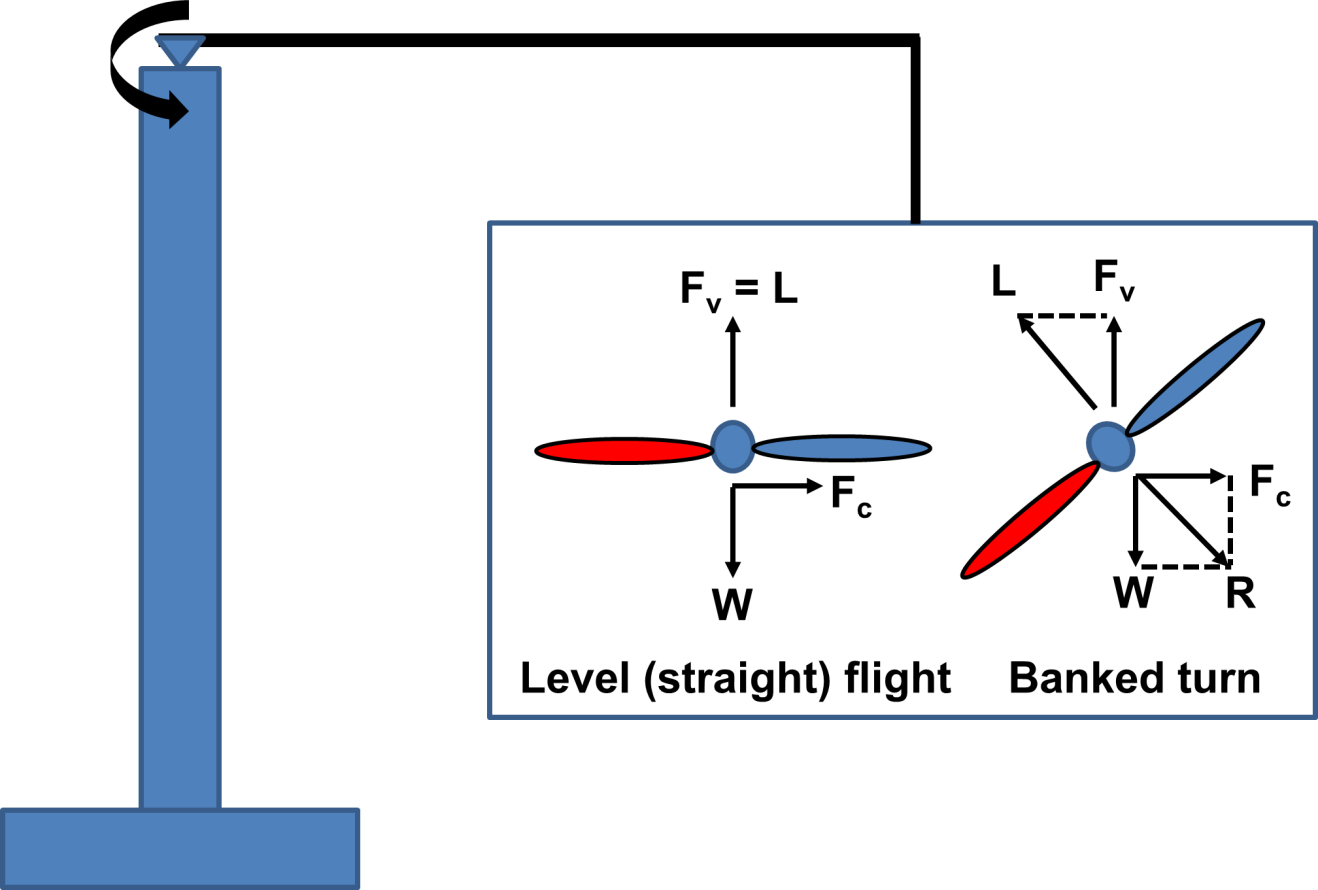
Force balance during banked and level flight in a circular trajectory. For a beetle flying in the level orientation (left) the lift force (L) acts vertically to counteract the weight (W) of the beetle. However, the beetle experiences a centrifugal side force (F_c_) due to the circular trajectory. For a beetle flying in the banked orientation (right) the lift force can be tilted towards the center of the circular trajectory to balance the resultant (R) of both the weight and centrifugal force. The wing colored red denotes the wing internal to the circular trajectory (closer to the center of the circle).

First, to fly in a flight-mill the insect needs to provide additional thrust (horizontal force) to overcome the additional air resistance and friction on the moving parts of the flight-mill. State of the art flight-mills use magnetic bearings to minimize friction (e.g. [14–15]). Nevertheless, air resistance to motion must be higher during flight in flight-mills compared to free (untethered) flight at the same speed. This implies that more flight "fuel" is used per distance covered in flight-mills [14,16]. Second, the rotation of the flight-mill represents only the horizontal force (thrust) applied by the insect. The flight-mill provides the vertical support for the insect’s weight whether it is flying or not. Thus, the energy expended to provide lift equivalent to body weight during normal flight (induced power) is not necessarily invested by tethered insects in flight-mills [13]. This may lower the energetic cost of flight in flight-mills, compared to free-flight, i.e, negating the added cost to overcome the resistance of the flight-mill. Alternatively, the insect in the flight-mill may be generating lift or side forces in excess of its body weight, as in take-off and climb. This extra vertical force would also not be accounted for by the rotation of the flight-mill, since the excessive vertical force would act against the rigid structure of the flight-mill. The uncertainty in lift produced during tethered flight thus makes it difficult to interpret the effort invested by the insect in the flight-mill. Third, in a flight-mill the insect is flying in a tight circle dictated by the length of the radial beam. Circular motion has a higher energetic cost than rectilinear motion at the same speed, primarily due to the increased drag associated with steering and the allocation of aerodynamic force to provide side forces to overcome the centripetal acceleration ([17] and Fig 1). However, in a flight-mill, the circular trajectory is forced by the device, and there is no way of determining whether the insect is steering or attempting to fly straight; and if steering, in what direction?

In most modern flight-mills the insect is rigidly tethered to the radial arm with the transverse axis of the insect parallel to the horizontal ground (e.g., [1,2–6]). This resembles the orientation for straight forward flight but precludes the swinging outwards of the insect that was so cleverly designed by Krogh and Weis-Fogh [11] for their roundabout. Consequently, the insect experiences an outwards side-force due to the centrifugal acceleration. Furthermore, because of the circular trajectory, the insect experiences an air flow typical of turning while its body orientation (roll) is typical of straight flight. How such inconsistency in sensory data affects flight-mill flight is not clear.

**Fig 2:**
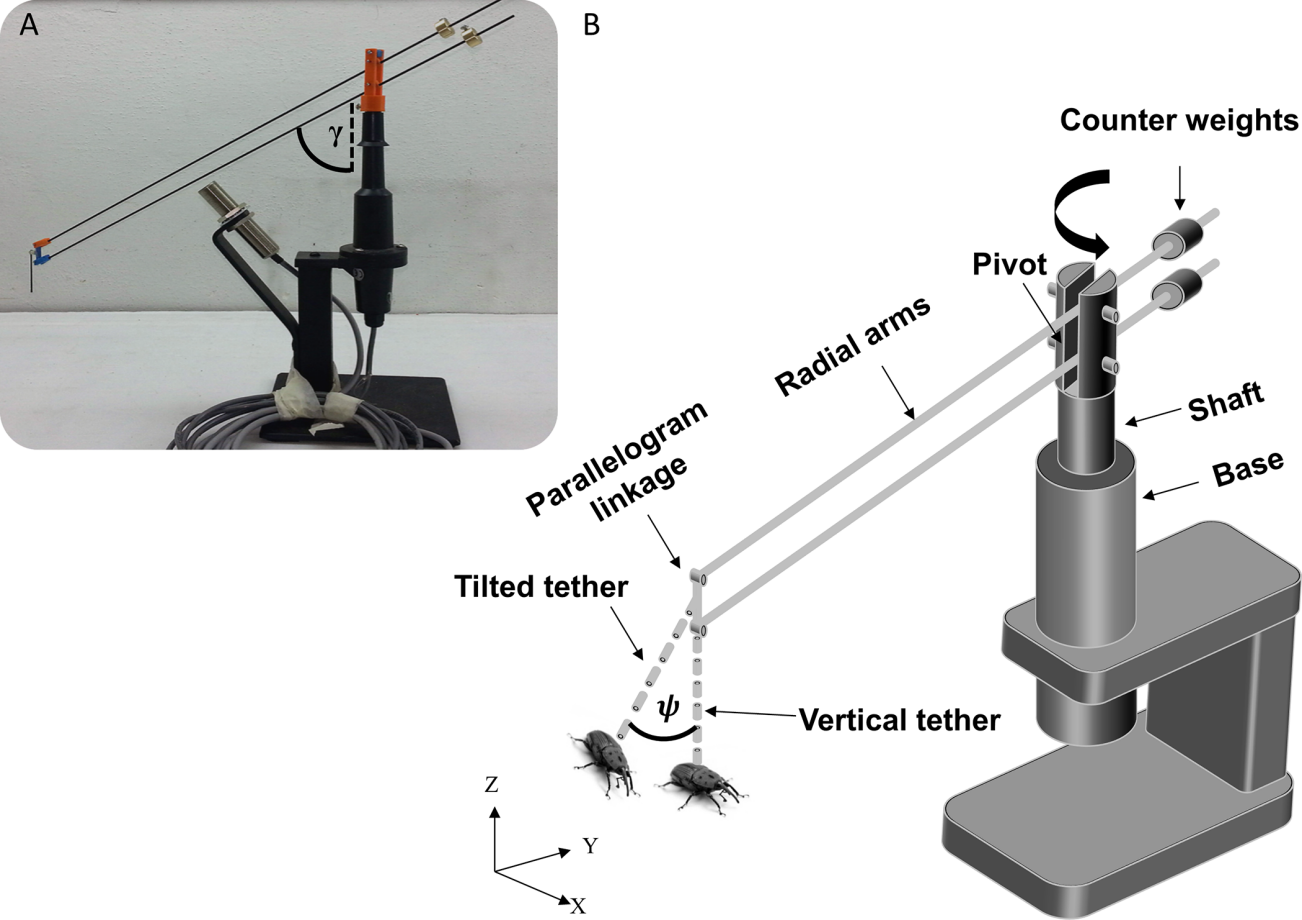
The flight-mill used in the study. The insert (A) shows a picture of the device and the definition of the elevation angle of the radial arm (*γ*) in the vertical plane. The schematic drawing (B) shows the various components and tethering orientation (level *versus* banked) of the insects. See text for a more elaborate description.

Here, we sought to evaluate how flight-mills affect the flight of the tethered insect. The red palm weevil (*Rhynchophorus ferrugineus*) is a competent flyer that readily makes prolonged flights in flight mills [1,3]. While attempts have been made to estimate dispersal flight distances from flight-mill studies, the relationship between flight-mill flight and free-flight is yet to be evaluated. Since the rotation of the flight-mill is provided by the flapping wings through the momentum imparted to air, the wing-beat kinematics holds the key to evaluating how flight muscle energy and aerodynamic force are invested during circular flight-mill flight. In this study, we addressed three basic questions in flight-mill studies: 1) Do the beetles in the flight-mill produce the lift needed to support their body weight in the air? 2) Does the circular trajectory result in wing-beat kinematics that is typical of steering; and if so, does tethering the beetles in a banked orientation rectify or strengthen steering attempts? 3) Do steering attempts or tethering angle affect the potential for prolonged flight as measured by flight-mills? To answer these questions, we conducted a comparative study in which we flew the same red palm weevils in four variants of the same flight-mill, while extracting the wing-beat kinematics using high-speed video cameras. The flight-mill variants allow to tether the beetles level or in a banked orientation (Fig 1) and the radial beam of the flight-mill can either be fixed to remain horizontal or made to pivot about the vertical shaft in the vertical plane (Fig 2 and description below). These variants were specifically designed to evaluate the lift production and the effect of body orientation on flapping kinematics.

## Methods

### Beetles

Red palm weevils, *R*. *ferrugineus*, were collected as pupae from infested palm trees in Israel. Post-emergence, weevils were maintained in jars containing bedding of coconut fibers and sugar cane (*ad-libitum*) as a food source. The beetles were housed in a temperature controlled (28° C) room with a light:dark cycle of 14:10 hrs. Because our study was focused on comparing flight-mill designs, only females were used in the study to prevent the possibility of inter-sex differences in flight kinematics interfering with the analysis. For the flight-mill experiments we selected the most active beetles, showing a high propensity for voluntary flight. The latter was evaluated by placing 5–7 beetles together, in a transparent container (12 × 8 × 6 cm L × W × H) at direct sunlight and choosing for the experiment those that voluntarily took-off within 2–3 minutes. All experiments were conducted in the summer at a room temperature of 27°C under bright light.

### Flight-mills and experiment design

The four variants of the flight-mills in our study (described below) are based on the flight-mill shown in Fig 2. For the vertical shaft with low friction we used the base of a wind sentry (Young, model 03102). The radial arm to which the beetles were tethered comprised two parallel, lightweight rods (diameter = 2mm each) made of carbon-fiber. The span of the radial arm from the tip where the insect was tethered to the shaft forming the vertical axis of rotation was 0.4 m. The beam extended an additional 0.13 m beyond the vertical shaft to support two 12 g counterweights made of brass. By carefully securing the counterweights at the right distance from the axis of rotation (typically 7.5 cm) the counterweights exactly balanced the weight of the contralateral side of the radial beam (to which the insect was tethered). The connection point of the radial arm to the vertical shaft was mounted on a pivot that allowed rotation in the vertical plane so that the two ends of the radial beam balanced as on a balance scale. When the insect was tethered to the longer end of the radial arm its body weight shifted the balance. Consequently, the arm rotated in the vertical plane to lower the insect towards the ground. When flight commenced the arm was elevated due to the lift provided by the insect and the centrifugal force resulting from the rotation of the flight-mill. The radial arm and the pivot point were linked in a 4-bar parallelogram so that as the arm end was lowered or elevated, the orientation of the transverse axis of the tethered insect (its roll angle, i.e., the rotation about the long axis of the body) remained the same. The insects were tethered by gluing the end of a small (5 cm long) metal pole (diameter = 1.5 mm) to their mesothoracic tergum using hot glue. The other end of the pole had a small connector that could be connected to the flight-mill either vertically or tilted (angle ψ in Fig 2B). We thus attached the beetles once with their lateral axis horizontal (hereafter ‘level’ flight condition) and a second time with their lateral axis rolled by 40° relative to the horizontal plane (‘banked’ condition, see Fig 1). We also filmed the same beetles under the banked and level conditions and in the same flight-mill, but this time after fixing the pivot point so that the radial arm remained horizontal regardless of the flight speed and flapping motions of the insects. We termed this flight-mill variant the ‘fixed’ pivot condition as opposed to the ‘seesaw’ condition when the beam was balanced on the pivot point. Consequently, each beetle (n = 10) was flown four times in each of the banked/level and fixed/seesaw combinations. The repeated measurements design of the experiment (40 flights by 10 beetles) allowed us to isolate the effect of flight-mill type from variance between individuals due to physiological condition, age or other unrelated factors.

In each trial, we first allowed the beetle to fly for approximately one minute to ensure steady flight before capturing their flight in the mutual field of view of three high-speed cameras (described below). We then stopped the beetles, adjusted the flight-mill to the new test condition, waited for flight to commence, allowed one minute of flight and recorded flight with the cameras. We consider it extremely unlikely that fatigue could have affected our results because other palm weevils had routinely flown in the same flight-mill for durations of up to 2 hours (see Results). In addition, the beetles rested for at least 2 minutes between the short trials. Nevertheless, to avoid measurement bias we systematically changed the order of the flight-mill trials for each beetle. The procedure was repeated until we had four recorded flights per each ten beetles (40 flights in total). Following the trials, the beetles were sacrificed and their body mass was measured to the nearest 0.1 mg with a digital scale (Boeco, model BAS 32 plus). All beetles flown had both of their wings intact. One wing of each beetle was removed to measure wing length, wing area and the second moment of wing area, as in [18]. The measurements of one wing were assumed applicable for the contralateral wing as well (I.e. assuming perfect bilateral asymmetry).

### High-speed recording and extraction of wingbeat kinematics

**Fig 3:**
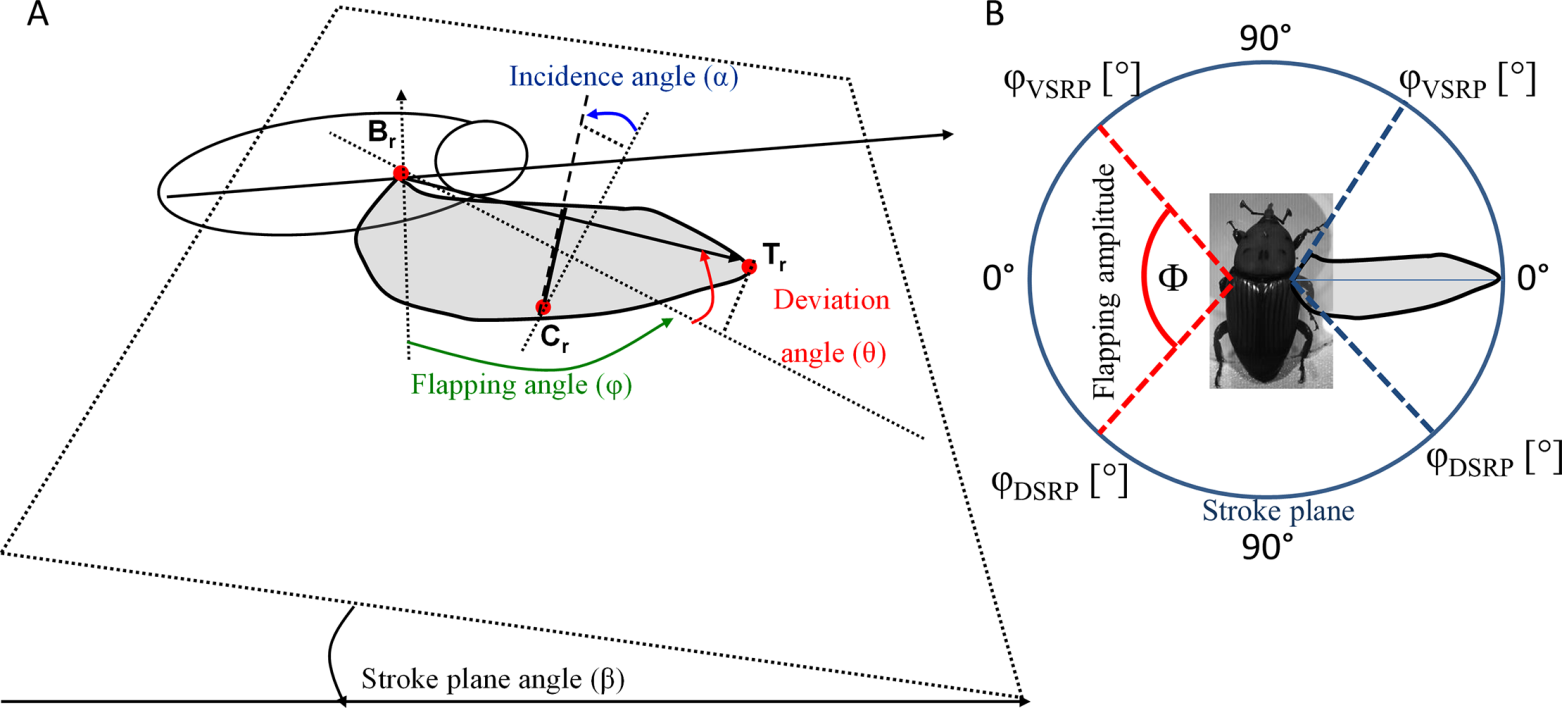
Definition of flapping kinematics. The area of the right wing is shown in grey. A) The motion of the wing tip relative to the body defines a stroke plane angle (β) in which the instantaneous flapping angle (*φ*) can be measured from the projection of the wing length onto that plane. The deviation angle is the deviation of the wing length from that plane. The geometric angle of incidence (α) is defined as the angle of the wing chord with the stroke plane. See appendix A for detailed explanation and definitions. B,T, and C are the wing base, wing tip, and a point on the trailing edge of the wing. The subscript “r” denotes that the points are on the right wing. B) The flapping motion of the wings in the stroke plane (blue circle). Dashed lines Illustrate the definition of flapping angles at the ventral and dorsal stroke reversal points (VSRP and DSRP, respectively). The angle between these two angular positions (Φ) is defined as the flapping amplitude.

The flight-mill was positioned so that a part of the circular trajectory transected the mutual field of view of three high-speed cameras (Fastcam SA3, Photorn Inc.), enabling simultaneous views of the flying beetle and the two wings from three different view-points. The cameras filmed at 2,000 frames per second (~20 video frames per flapping cycle) and were spatially calibrated [19]. In each video frame of two consecutive flapping cycles we determined the positions of the following points on the body: the point of attachment to the tether (P), the left (B_l_) and right (B_r_) wing bases, the left (T_l_) and right (T_r_) wingtips, and two points (C_l_, C_r_) where the cubitus intersects the trailing edge of the left and right wings, respectively (Fig 3A). The change in position of the point of attachment to the tether with time (between video frames) was used to determine the instantaneous flight velocity as in Rayner and Aldridge [20]. The remaining points were used to extract the wingbeat kinematics of the left and right wings as three (per wing) time varying angles (flapping, deviation, and incidence, see Fig 3) as defined by Ellington [21] and Fry et al. [22,23]. In each trial we extracted the kinematic data from two consecutive flapping cycles and averaged the kinematic data from both cycles. From the data we calculated the following kinematic parameters: 1) flapping frequency, 2) the stroke plane angles of both wings, 3) flapping amplitude, 4) the angular position of the wings in the dorsal, and ventral (5) stroke reversal points (Fig 3B), 6) the geometric angle of incidence of the wing, relative to the stroke plane (Fig 3A) during the mid-stroke of the upstroke and downstroke, and 7) the average wingtip speeds of the wing relative to air during the upstroke and downstroke. The calculations used to extract these parameters are described in Appendix A.

### Measurement of flight force from the flight-mill dynamics

In the seesaw condition, the flight-mill operates as a compound conical pendulum so that, with the known mass of the beetles, flight speed and angle of the radial arm in the vertical plane (*γ*, in Fig 2), the vertical aerodynamic force generated by the beetle can be estimated. The calculation is detailed in Appendix B. Here only the end result measuring the aerodynamic up-thrust is given:

$$F_{Va}=-\frac{\omega^{2}\left[\sin\gamma \;\cos\gamma \left(l_{2}^{2}m_{2}+l_{1}^{2}m_{1}\right)+l_{3}^{2}m_{3}\sin\left(\gamma -\vartheta \right)\cos\left(\gamma -\vartheta \right)\right]-m_{3}gl_{3}\sin\left(\gamma -\vartheta \right)}{l_{3}\sin\left(\gamma -\vartheta \right)}$$ (1)

where *ω* is the angular velocity of the flight-mill; *l*_1_, *l*_2_, and *l*_3_ are the distances from the pivot to the center of mass of *m*_1_, *m*_2_, and *m*_3_, which in turn are the mass of the long end of the radial arm (to which the insect is tethered), the short end of the arm (with counterweights) and the beetle, respectively. The angle *ϑ* is a correction angle equal to 6.5° and 4.5° when the beetles are in the level and banked orientation, respectively. This accounts for the lower position of the beetle relative to the end of the radial arm. The force *F*_*Va*_ is the extra vertical force needed to explain the balance of forces of the pendulum in the vertical plane. For the level beetles *F*_*Va*_ is lift, while for the beetles in the banked position (banking angle *ψ* = 40°) the lift is expected to be *F*_*Va*_/cos *ψ*.

The horizontal flight force was measured from the force needed to maintain the flight-mill rotating at constant speed. This force was determined by briefly rotating the flight-mill in the fixed condition, and then measuring its resistance to rotation (resistance torque) from its angular deceleration. The angular deceleration and rotation speed of the flight-mill were measured by filming the experiment with the high-speed cameras and determining the first and second time derivatives of the position of the tip of the flight-mill in consecutive video frames. This was performed once with a flightless beetle tethered to the flight-mill and again without the beetle. Data from the different trials were pooled to obtain the general relationship between rotation speed and deceleration. The relationship between the resistance torque (*τ*), which slows down the rotation of the flight-mill, and the angular deceleration of the flight-mill ($\dot{\omega }$) is:

$$\tau =I_{zz}\dot{\omega }$$ (2)

where *I*_*zz*_ is the mass moment of inertia of the flight-mill in the fixed condition for rotation about the vertical shaft. The mass moment of inertia (*I*_*zz*_) was measured from the mass and geometry of the different components of the flight mill to be 9.709×10^−4^ and 8.108×10^−4^ kg m^2^ with and without the flightless beetle, respectively.

For the flight-mill to rotate at a constant speed the beetle must generate torque to overcome the resistance torque. From the attachment point of the beetle at the end of the radial arm the horizontal aerodynamic force needed (*F*_*Ha*_) is:

$$F_{Ha}=\frac{\tau }{l}$$ (3)

where *l* is the length of the radial arm (0.4 m).

### Force estimation from wingbeat kinematics

The wingbeat kinematics provide a description of how flapping varied between the different flight-mills variants. To evaluate the joint effect of changes in flapping kinematics on flight, we calculated the quasi-steady aerodynamic forces from wing translation relative to air using the simplified blade-element model [24]. The simplified model assumes that the flapping wings are rigid, while ignoring rotational, added mass, and inertial forces (for a description of these other forces see [25]). For an elaborated description of the calculations and assumptions see Appendix C. In brief, the flapping speed of each wing was found in the body frame of reference. We then used vector summation to add the forward flight speed of the insect and obtained the speed of the wings relative to stagnant air. The angle of attack was defined as the geometric angle of incidence between the wing chord and the direction of wing motion relative to air. We then estimated the resultant aerodynamic force from the instantaneous lift *L*_(*t*)_ and drag *D*_(*t*)_ forces:

$$L_{\left(t\right)}=\frac{1}{2}\;\rho AC_{L\left(\alpha \right)}{\left({\hat{r}}_{2}U_{w\left(t\right)}+U_{f}\right)}^{2}$$ (4)

$$D_{\left(t\right)}=\frac{1}{2}\;\rho AC_{D\left(\alpha \right)}{\left({\hat{r}}_{2}U_{w\left(t\right)}+U_{f}\right)}^{2}$$ (5)

where *ρ* is air density at room temperature and sea level (taken to be 1.2 kg m^-3^), A is the area of the wing, ***U***_***w***(***t***)_ is the instantaneous flapping velocity of the wingtip in the sagittal plane, ${\hat{r}}_{2}$ is the non-dimensional radius of the second moment of wing area, as defined by Ellington [26], and ***U***_***f***_ is the forward (flight) velocity. We used different ***U***_***f***_ values for the left and right wings according to their distance from the axis of rotation of the flight-mill. The force coefficients *C*_*L*(*α*)_ and *C*_*D*(*α*)_ and their relationship with the angle-of-attack of the wings were generalized from published data on flapping insect wings. The aerodynamic force was deconstructed into its horizontal and vertical components, giving the forces available to rotate the flight-mill and provide weight support, respectively.

### Prolonged flight at the level and banked orientation

Our findings suggested that the orientation of the beetles in the flight-mill affected their flapping kinematics (see below) without affecting flight speed. Hence, we hypothesized that body orientation can lead to changes in flight efficiency, impacting the energetics of prolonged flights. To test this hypothesis, we used the flight-mill in the fixed condition and tethered a naïve group of female beetles either at the level body orientation or at the banked orientation. The feeding regime of the beetles prior to the experiment was *ad-libitum*. Each beetle was tethered for two hours and encouraged to fly (see below) continuously during this time period. The body mass of the beetles was measured before the trial and after two hours on the flight-mill. Whenever a beetle stopped flying we used tactile and air flow stimuli to induce it to resume flight. Thirty beetles were tested on the same flight-mill at the level flight orientation and 31 at the banked orientation. An additional 13 beetles were attached to the flight-mill for two hours without flying (they were not stimulated to fly, and if flight initiated spontaneously we arrested it by touching the tarsus of the beetle). These provided the baseline for mass loss during two hours while at rest. After the 2-hr trials, the reduction in body mass was converted to % of initial body mass, and the mean of the control group was subtracted from the % reduction in body mass of each beetle in the flight groups. We then compared mass reduction between the two groups, once using the distance flown and again using the actual (out of the 2-hr session) time flown as covariates. Since the dependent variable was a proportion (%), the data were arcsine transformed prior to ANCOVA testing.

### Statistical analysis

Each of the ten weevils in the study was flown in the four variants of the flight-mill, and thus provided all possible combination of roll and pivot. For general flight data (flight speed and wingbeat frequency) we analyzed the data using a 2-way factorial analysis for repeated measurements (RMANOVA) with orientation (i.e. beetle banked versus level) and pivot (seesaw versus fixed horizontal) as the two factors. For the wingbeat kinematics data, we used a 3-way factorial analysis for repeated measurements adding wing side (left and right wing) as another factor. All analyses were performed with Statistica (v12, StatSoft, Inc.). General Linear Model was applied with all interactions included in the model design and confidence level set to 95%. Tukey post-hoc tests were used for exploration of significant interactions. Means are reported ± one standard error (SE), and unless otherwise specified the sample size is n = 10 weevils.

### Free-flight in circles

To evaluate if the circular flight trajectories of flight-mills can be achieved during free-flight we filmed another set of female beetles flying within a 4 x 4 x 3 m room. A lamp at the center of the room attracted the beetle to maneuver around it (Pers. Obs.) making curved trajectories that were tracked using three-high speed cameras. The cameras were spatially calibrated as described above but due to the larger spatial scale we could only analyze the flight trajectories. We were able to obtain circular flight data for 13 beetles (all females). For each flight trajectory we measured the speed of flight, the curvature of the flight trajectory, and the centripetal acceleration as in [27]. These were compared to the same parameters from the flight-mill flight.

## Results

**Fig 4:**
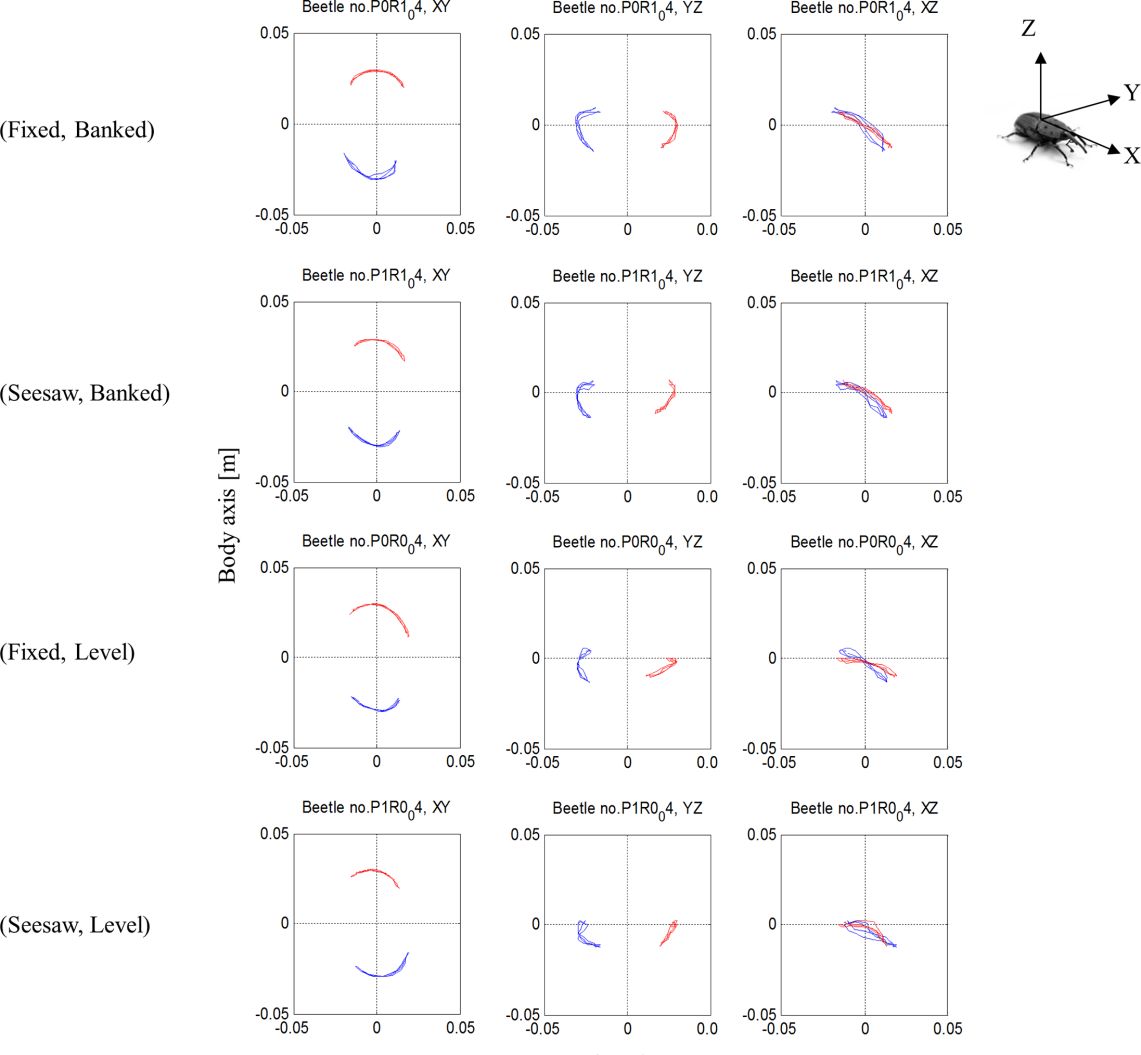
Wingtip trajectory in the body frame of reference during flight in the flight-mill. The rows present the four flights by the same beetle (beetle #4) in the four variants of the flight-mill. The trajectories of the left and right wingtips are denoted by red and blue color respectively. The left wing is the wing internal to the circular flight trajectory. Left, center, and right columns are the trajectory of the wingtips in the dorsal (XY), transverse (YZ), and sagittal (XZ) planes respectively, where X,Y,Z are the body axes defined in the insert on the right. The horizontal axis in each graph is the X, Y and X body axis and the vertical axis is the Y, Z and Z body axis in the left, center, and right column, respectively. The origin is always the base of the wing and all units are meters.

Table 1 presents the morphological measurements of the 10 beetles used in the study. The flight speed of the beetles (mean = 1.85 ± 0.07 m s^-1^) did not differ significantly between banked and level beetles (RMANOVA, F_1,9_ = 3.5, p = 0.094) or between the fixed and seesaw design (RMANOVA, F_1,9_ = 1.42; p = 0.263) and neither did wingbeat frequency (RMANOVA, F_1,9_ = 2.5, p = 0.147 and F_1,9_<0.01, p = 0.981, respectively, mean = 96.8 ± 0.67 Hz). In contrast, the wingbeat kinematics of the beetles varied among the four flight-mill variants and the wingbeat kinematics of the left wing varied significantly from the right wing within the same flight-mill (Fig 4). These differences are described below and summarized in Table 2.

**Table 1:** Body mass and wing measurements of beetles used in the study.

*Beetle number*	*Body mass [g]*	*Wing length [mm]*	Area of the wing pair^*^ *[mm*^*2*^*]*	*r*_2_^**^	*Aspect- ratio* ^***^
#1	1.071	22.53	210.24	0.547	9.7
#2	1.139	25.17	257.18	0.548	9.8
#3	1.070	22.28	200.85	0.552	9.9
#4	0.977	22.53	212.54	0.553	9.5
#5	0.925	21.78	196.04	0.543	9.7
#6	1.222	23.52	240.17	0.547	9.2
#7	0.855	20.96	177.61	0.546	9.9
#8	1.185	22.86	224.9	0.545	9.3
#9	1.081	23.52	249.3	0.535	8.9
#10	1.084	23.68	237.27	0.553	9.5
Mean ± SE	1.061 ± 0.035	22.9 ± 0.37	220.6 ± 8.07	0.547 ± 0.002	9.5 ± 0.33

* the measured area of one wing × 2

** *r*_*2*_ is the non dimensional radius of the second moment of wing area (Ellington 1984)

*** Aspect-ratio is the ratio between the span and mean chord of the wings

**Table 2:** Repeated measures ANOVA results (p-values) showing the significance of the effect of wing side (left /right), tethering orientation of the beetle (level/banked), and pivot (fixed/seesaw) of the radial beam on flapping kinematics. All three-way interactions were not statistically significant (P>0.05).

*Dependent variable*	*Wing side (left/right)*	*Banking (banked/level)*	*Pivot (fixed/seesaw)*	Side*Banking	Side*Pivot
Flight speed	-	0.094	0.263	-	-
Wingbeat frequency	-	0.147	0.981	-	-
Stroke plane angle (β)	0.061	**0.004**	0.691	0.0623	0.173
Flapping amplitude (Φ)	**0.036**	0.761	0.208	0.336	0.706
DSRP (φ_max_)	0.523	**0.011**	0.077	**0.005**	**0.033**
VSRP (φ_min_)	0.275	**<0.001**	0.558	0.283	0.400
Incidence Downstroke	0.070	**0.013**	0.978	0.962	0.373
Incidence Upstroke	**<0.001**	0.294	0.073	**0.015**	0.548
Wing tip speed Downstroke	0.538	0.735	0.441	0.351	0.930
Wing tip speed Upstroke	**<0.001**	0.316	0.522	0.398	0.480

### The effect of pivoting the radial arm

**Fig 5:**
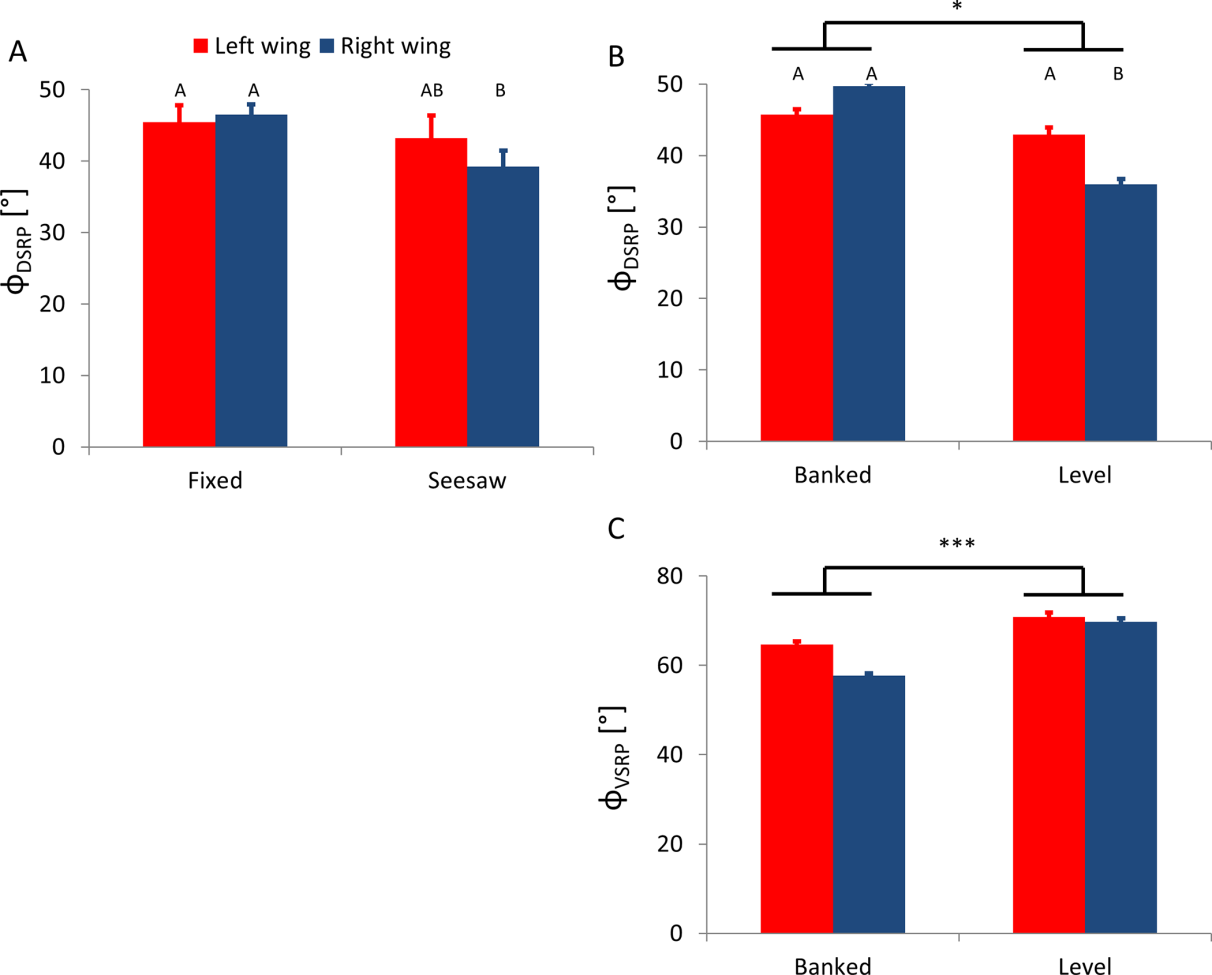
Changes in angular positions of the stroke reversal points in the different variants of the flight-mill. Red and blue colors denote the left (internal) and right wings, respectively. Asterisks denote significance of the fixed/seesaw (A) or bank/level (B,C) effects reported by a repeated measurements ANOVA (* p<0.05, ** p<0.01, *** p<0.001). Capital letters denote significant statistical differences (P<0.05) and similarities in a Tukey post-hoc test when the interaction with wing side is significant. A) The dorsal stroke reversal point of the right wing in the seesaw condition was significantly lower (less dorsal) compared to the left or right wing when the flight-mill was in the fixed condition (Tukey, p<0.005 in both cases). B) The dorsal stroke reversal point was higher in the banked condition compared to the level condition (F_1,9_ = 10.1, p = 0.011). A significant interaction between banking condition and wing side (F_1,9_ = 13.5, p = 0.005) revealed that the right wing during leveled flight reached lower (less dorsal) dorsal stroke reversal angles than the left wing (Tukey, p = 0.033) and lower angles than either the left or right wings during flight in the banked orientation (Tukey, p<0.008). C) The ventral stroke reversal point was significantly lower (more dorsal) in the banked condition (F_1,9_ = 23.5, p<0.001).

The freedom of the radial beam to move up and down in the vertical plane (seesaw condition) had a weakly significant effect only on the dorsal stroke reversal point through an interaction with wing side (RMANOVA, F_1,9_ = 6.35, p = 0.033, Table 2): namely, the effect of pivot type on the DSRP was inconsistent for left and right wings (Fig 5A).

### Banked orientation effect

**Fig 6:**
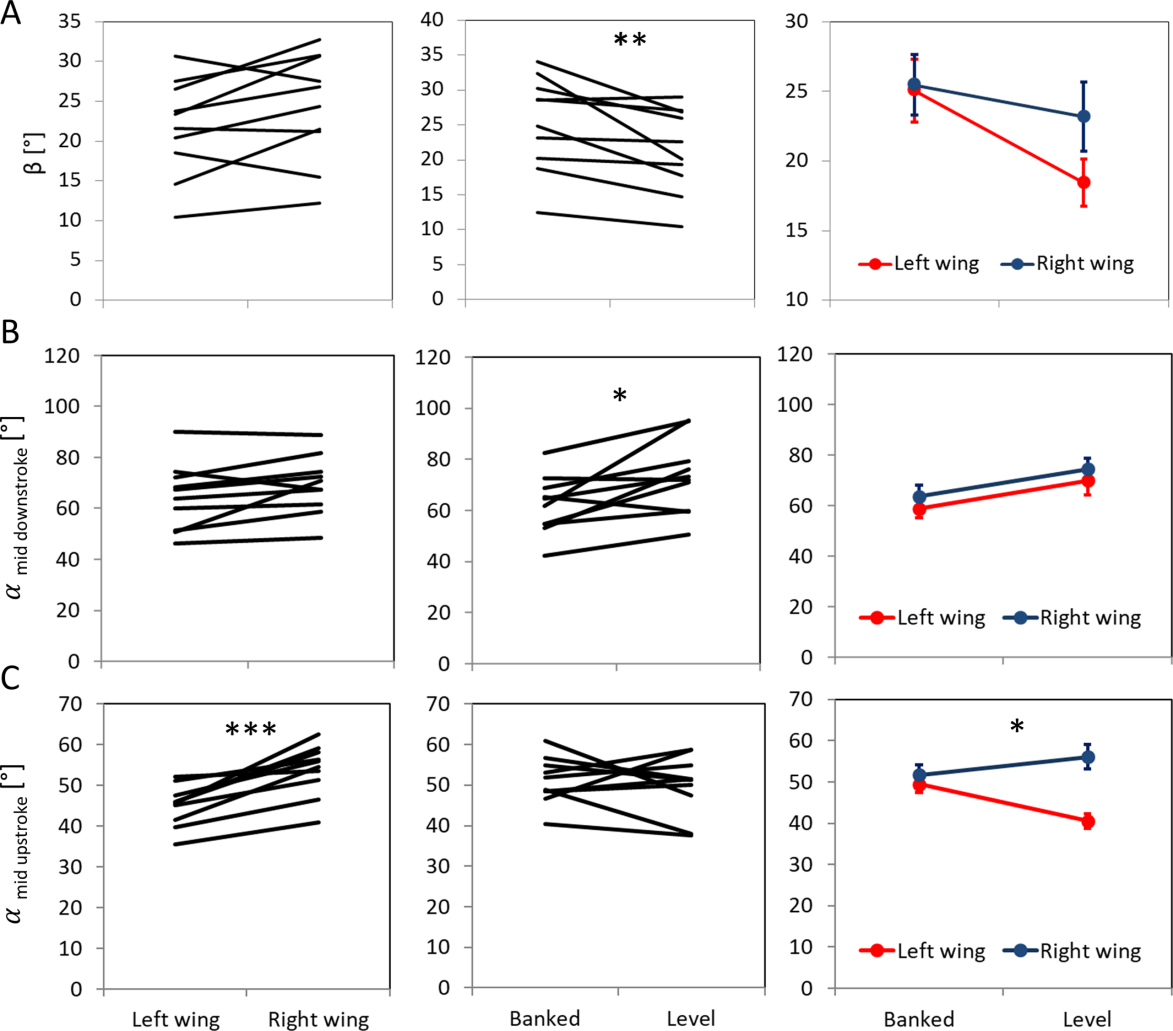
Effect of wing side and body orientation on flapping kinematics I. The left and center columns show the change between the left and right wing and the level and banked flight orientation, respectively, for each beetle (black lines). The right column presents the interaction between wing side and body orientation. Significance is denoted by asterisks as in Fig 5. A) The Stroke plane angle (β) was significantly larger when the beetles were banked (RMANOVA, F_1,9_ = 14.6, p = 0.004) B) Geometric angle of incidence (α) during mid downstroke was not significantly different between the left (internal) and right (external) wings (RMANOVA, F_1,9_ = 4.2; P = 0.070). The angles of incidence were significantly smaller when the beetles were flying banked compared to level (F_1,9_ = 9.5; p = 0.013). C) Geometric angle of incidence during mid upstroke was significantly smaller in the left compared to the right wing (RMANOVA, F_1,9_ = 34.6, p <0.001 and this bilateral flapping asymmetry was larger when the beetles were level (F_1,9_ = 8.9, p = 0.015 See Fig 3A for definitions of these angles.

**Fig 7:**
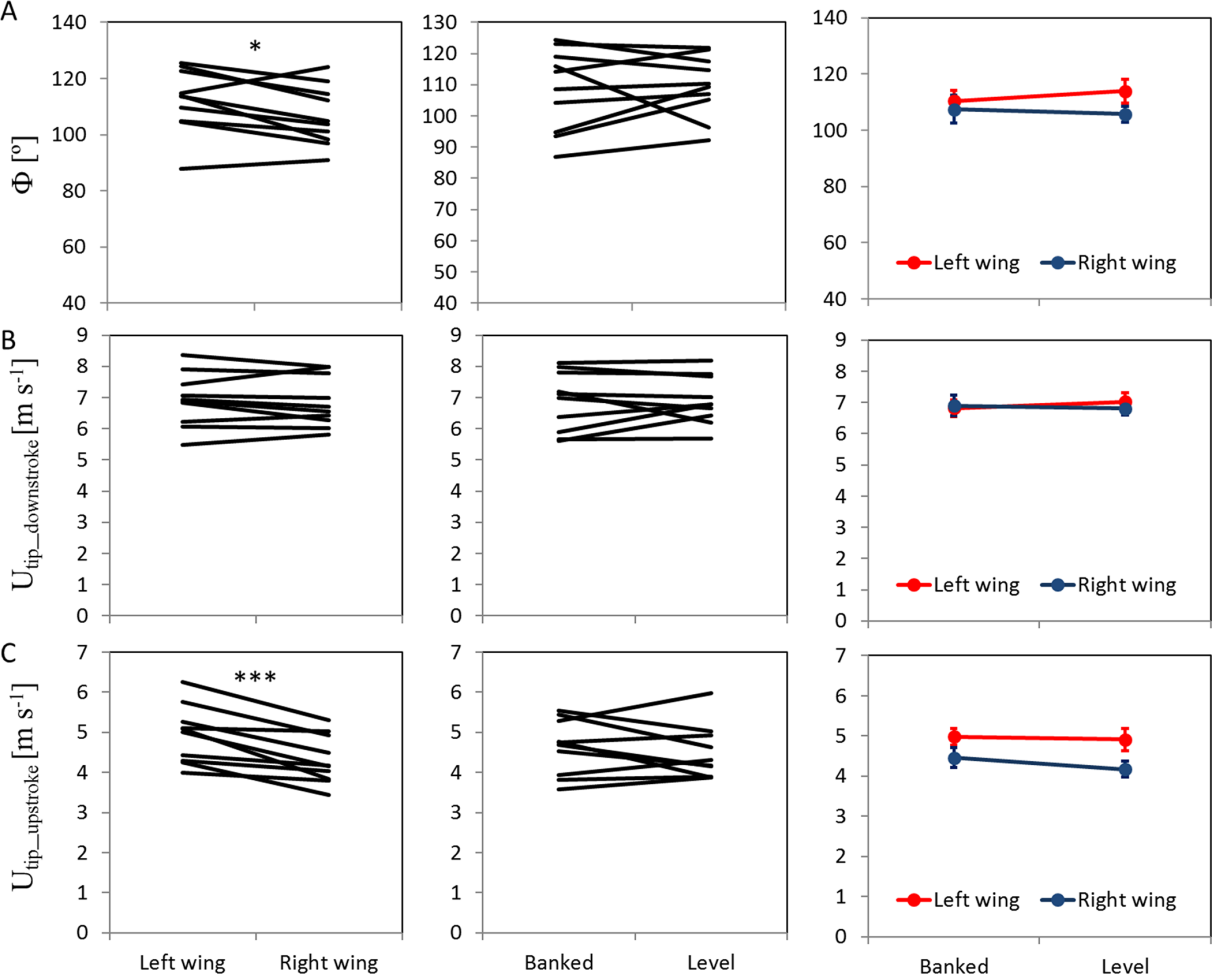
Effect of wing side and body orientation on flapping kinematics II. The figure is arranged as in Fig 6. A) Flapping amplitude was significantly higher in the left wing. B and C) mean speed of the wingtip relative to air during the downstroke and upstroke, respectively. The speed of the left wing was significantly higher than that of the right wing during the upstroke.

When the beetles were rotated into the banked turn orientation, their dorsal and ventral stroke reversal points shifted dorsally (Fig 5B and 5C), their stroke plane angle was significantly steeper (RMANOVA, F_1,9_ = 14.6, p = 0.004, Fig 6A), and the angle of incidence of their wings was significantly smaller during the down-stroke (RMANOVA, F_1,9_ = 9.5, p = 0.013) compared to when the beetles’ orientation was level (Fig 6B). Despite the changes in angular position of the stroke reversal points, the flapping amplitude was not affected by banking (mean amplitude: 108.9 ± 3.9 and 109.7 ± 3.3° for the banked and level flight, respectively, RMANOVA, F_1,9_ = 0.098, p = 0.76, Fig 7A).

### Flapping asymmetry

The wing inside the circular flight path had a higher flapping amplitude (RMANOVA, F_1,9_ = 6.06, p = 0.036, Fig 7A), lower angle of incidence during the upstroke (RMANOVA, F_1,9_ = 34.6, p<0.001, Fig 6C), and a higher wingtip speed during the upstroke (RMANOVA, F_1,9_ = 25.91, p<0.001, Fig 7C) compared to the wing external to the circular flight path. There were also significant interactions between wing side and the orientation of the beetles (Table 2) in wing incidence during the upstroke (RMANOVA, F_1,9_ = 8.9, p = 0.015, Fig 6C) and the dorsal stroke reversal point (RMANOVA, F_1,9_ = 13.51, p = 0.005, Fig 5B). Namely, during the upstroke, the angle of incidence of the right (external) wing was higher than the left (internal) wing when the beetles were level (Tukey, P = 0.003). When the beetles were level the external wing also reached a lower dorsal stroke reversal point compared to the internal wing (Tukey, p = 0.034) and compared to the internal and external wings when the beetles were banked (Tukey, p<0.007 in both cases, Fig 5B).

### Force required to lift and rotate the flight-mill

**Fig 8:**
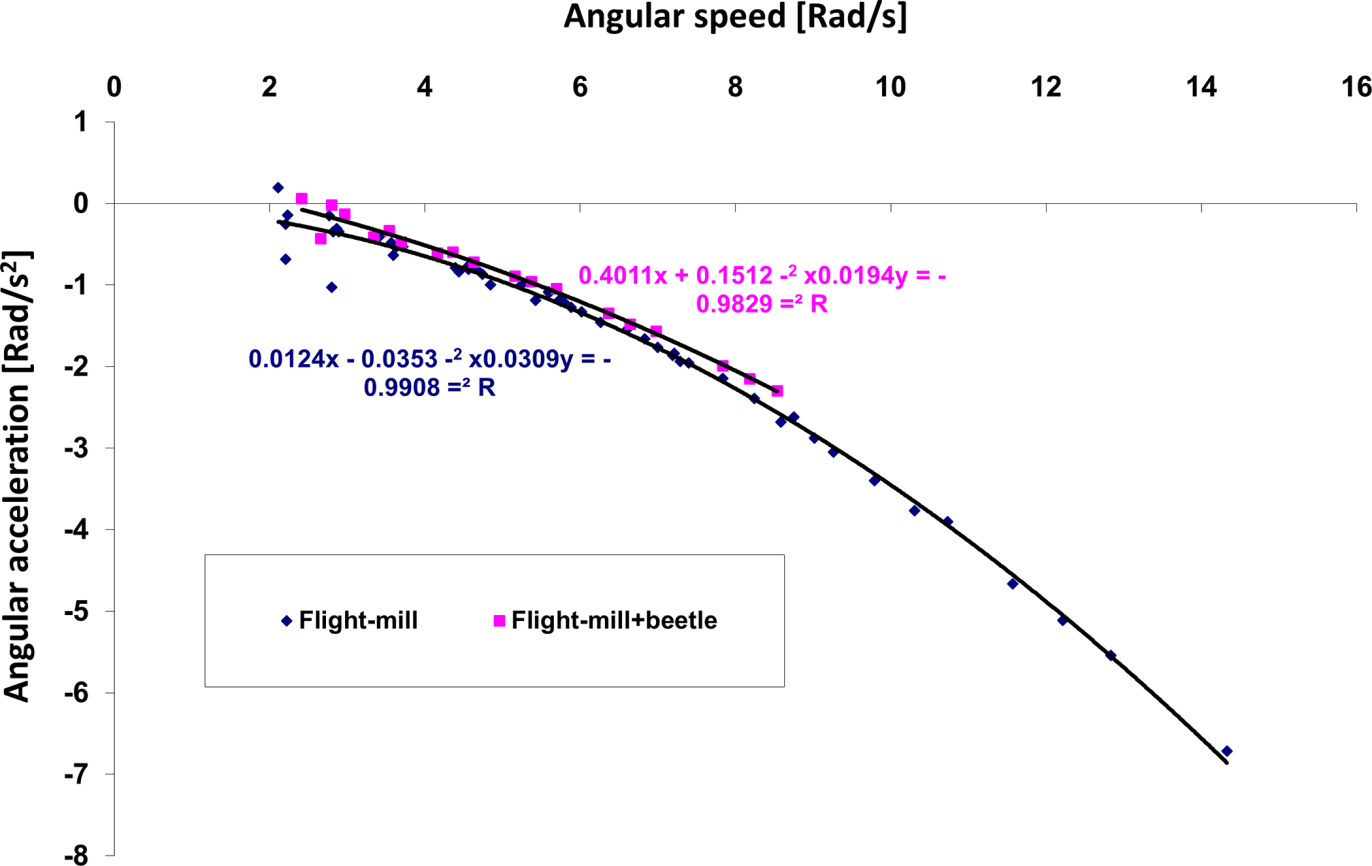
Empirical measurements of the relationship between turning rate (angular speed in radians per second) and angular deceleration (radians s^-2^) of the flight-mill. The deceleration is due to resistance of the flight-mill to rotation with and without a tethered flightless beetle. The relationship is used to estimate the horizontal force that the beetle must overcome to maintain the flight-mill rotating at a constant speed. The data shown are for the flight-mill in the fixed condition only.

To fly at constant speed, a beetle needs to provide extra thrust to counter the resistance torque (due to air resistance and friction) of the flight-mill. Fig 8 shows the relationship between the angular speed (turning rate) and the angular deceleration of the flight-mill in the fixed condition. For the average flight speed measured in our beetles (1.8 m s^-1^ = 4.65 rad s^-1^) the angular deceleration was 0.72 and 0.84 rad s^-2^, with and without a flightless beetle tethered to the end of the radial beam, respectively. Consequently, at the average flight speed observed, the minimum thrust needed is 1.75 mN, which is equivalent to 16.8% of average body weight (1.06 g, Table 1) of our beetles.

**Fig 9:**
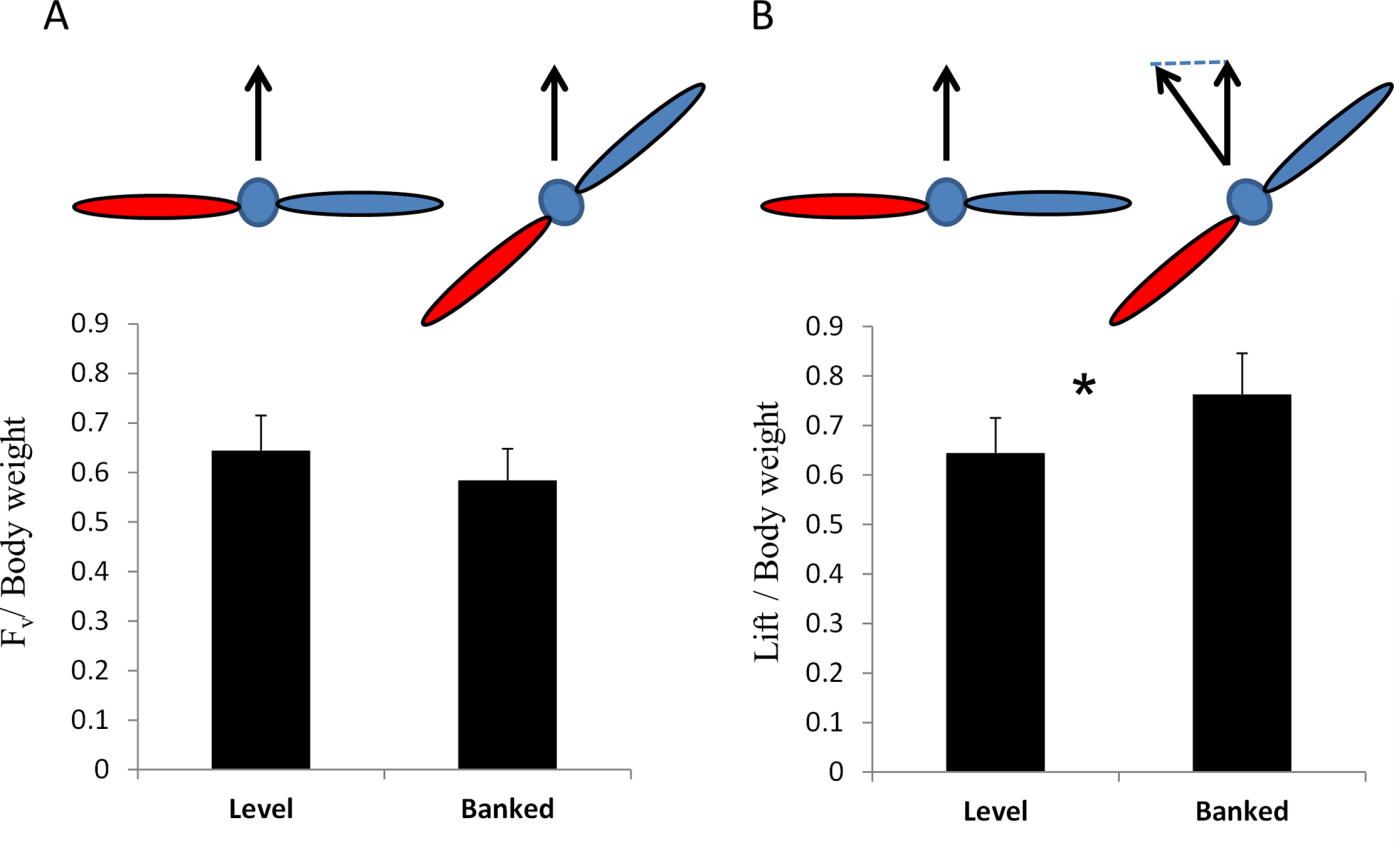
Aerodynamic vertical force generated by the tethered beetles. The forces are calculated from the conical pendulum analysis. A) the mean vertical force (F_v_) for the beetles in the level and banked orientation. Both forces are normalized by the beetle’s body weight B) The lift forces. Data as in (A) after correcting for the banking angle to give the force normal to the dorsal plane (lift). Red and blue colors denote the left (internal) and right wing, respectively.

From the pivot angle of the radial beam in the seesaw condition (*γ*, observed range: 72°- 85°) we found that the aerodynamic vertical force invested by the beetles in the seesaw design was 64% ± 7% and 58% ± 6% of their body weight when they were in the level and banked orientation, respectively (Fig 9). The remaining up-thrust to enable a horizontal flight trajectory came from torque due to centrifugal force (associated with the rotation of the flight-mill). Vertical force did not differ between the level and banked beetles (Paired t-test, t_9_ = 1.36, p = 0.2) but the resulting estimate of lift force (Fig 9B) for the banked beetles (76% ± 8% of body weight) was significantly higher than when the beetles were level (Paired t-test, t_9_ = -2.4, p = 0.035).

### Steering derived from flapping kinematics

**Fig 10:**
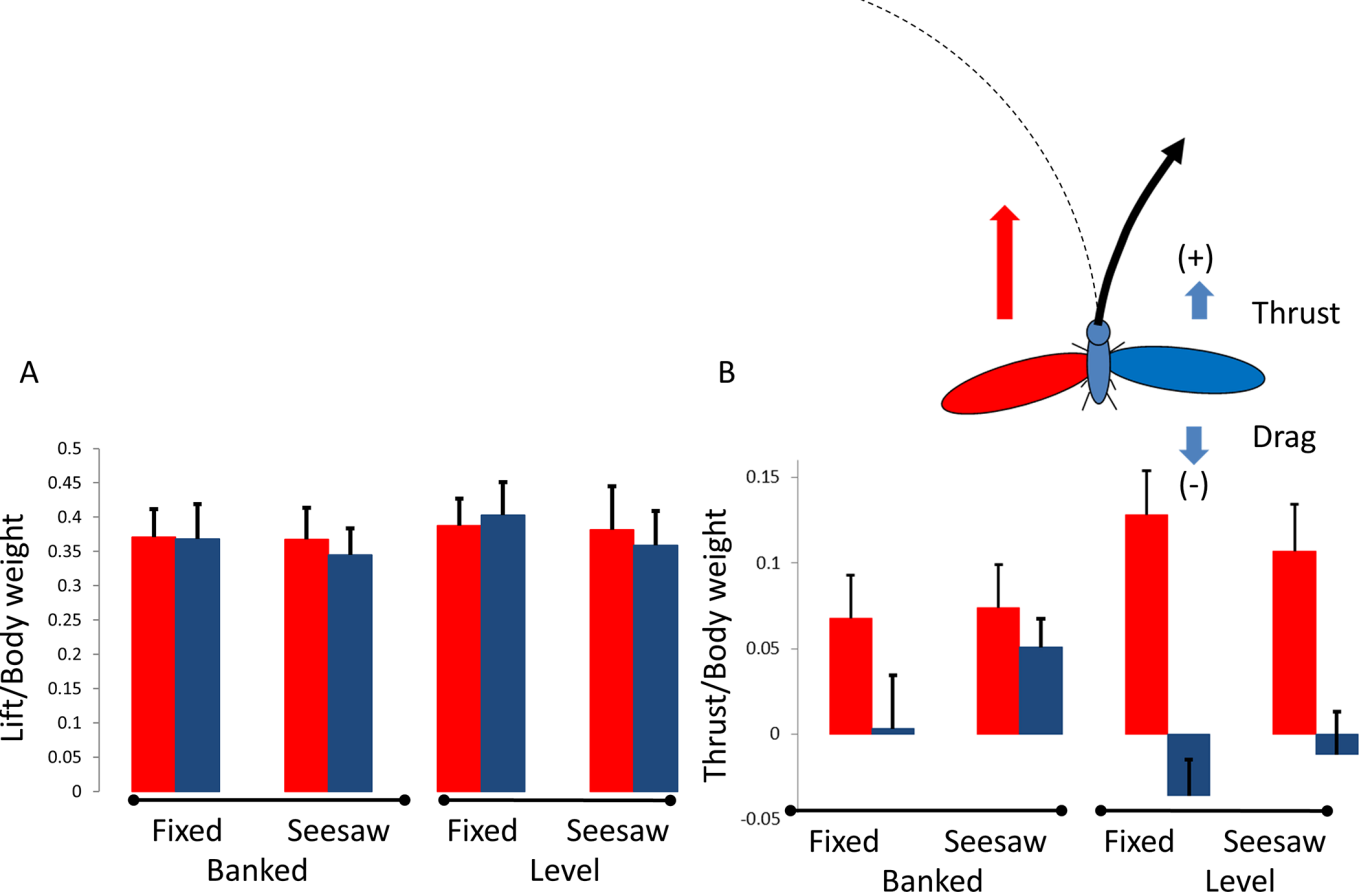
Aerodynamic forces estimated from the quasi-steady analysis. A) Lift forces. B) Horizontal (thrust) forces. Red and blue colors denote the left (internal) and right wing, respectively. Both forces are normalized with the beetles’ body weight. Thrust/drag is defined as positive and negative when directed forwards and backwards, respectively. The insert above B shows the yaw torque due to asymmetric flapping that should lead to exiting the circular flight trajectory in free-flying beetles.

The quasi-steady lift force estimated from the wingbeat kinematics of the same flight-mills in the seesaw condition were 71% ± 7.6% and 74% ± 11% of the body weight for the banked and level orientation, respectively (Fig 10). In the banked beetles the vertical component of lift amounted to 55% ± 5.9% of body weight. The estimates of lift force did not differ between the internal and external wings or between banked and level beetles (RMANOVA, F_1,9_ = 0.33, p = 0.58 and F_1,9_ = 0.97, p = 0.35, respectively). In contrast, the estimated horizontal forces were largely contributed by the internal wing (RMANOVA, F_1,9_ = 22.92, p<0.001, Fig 10) and a significant interaction between side and orientation (F_1,9_ = 9.06, p = 0.015) revealed that the thrust produced by the external wing was lower when the beetles were level compared to the contribution of the left (internal) wing when the beetles were banked (Tukey, p<0.001) or level (Tukey, p = 0.011).

### Indirect estimates of cost of flying in the level and banked orientation

**Fig 11:**
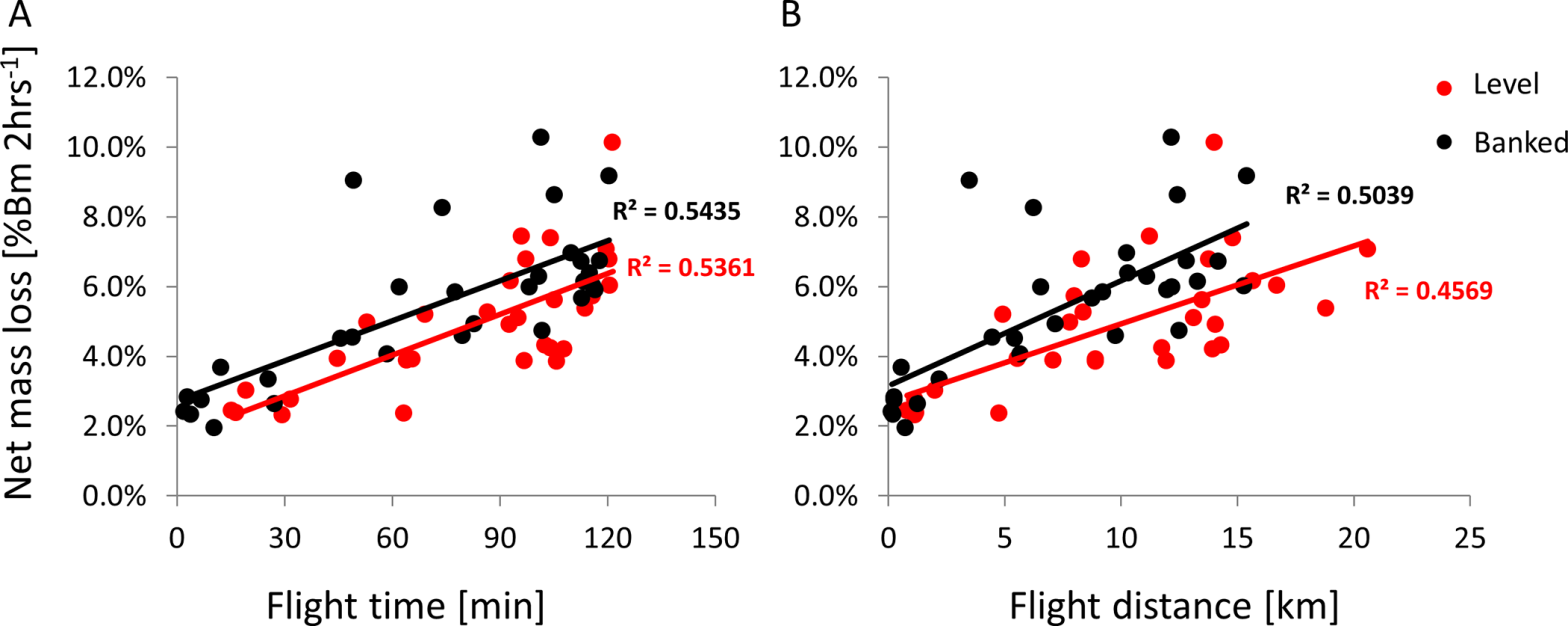
Effect of tethering orientation on mass loss during prolonged flight in the flight-mill. Each symbol denotes the % of net body mass lost due to flight during two hours of tethering to the flight mill. Black and red colors denote banked and level body orientation, respectively. A) Mass loss as a function of actual flight time. B) Mass loss as a function of flight distance.

Beetles lost 2.2% ± 0.22% (n = 13) of their initial body mass while tethered to the flight-mill for two hours without flying. In flying beetles, mass loss was highly variable, as was the propensity to fly for the entire duration of the trial. Nevertheless, mass loss increased with the distance and time flown (Fig 11), and ANCOVA revealed that banked beetles lost significantly more mass than level beetles when distance and time flown were used as covariates (F_1,58_ = 8.99, P = 0.004 and F_1,58_ = 7.35, P = 0.009 respectively).

### Free-flight in circles

**Fig 12:**
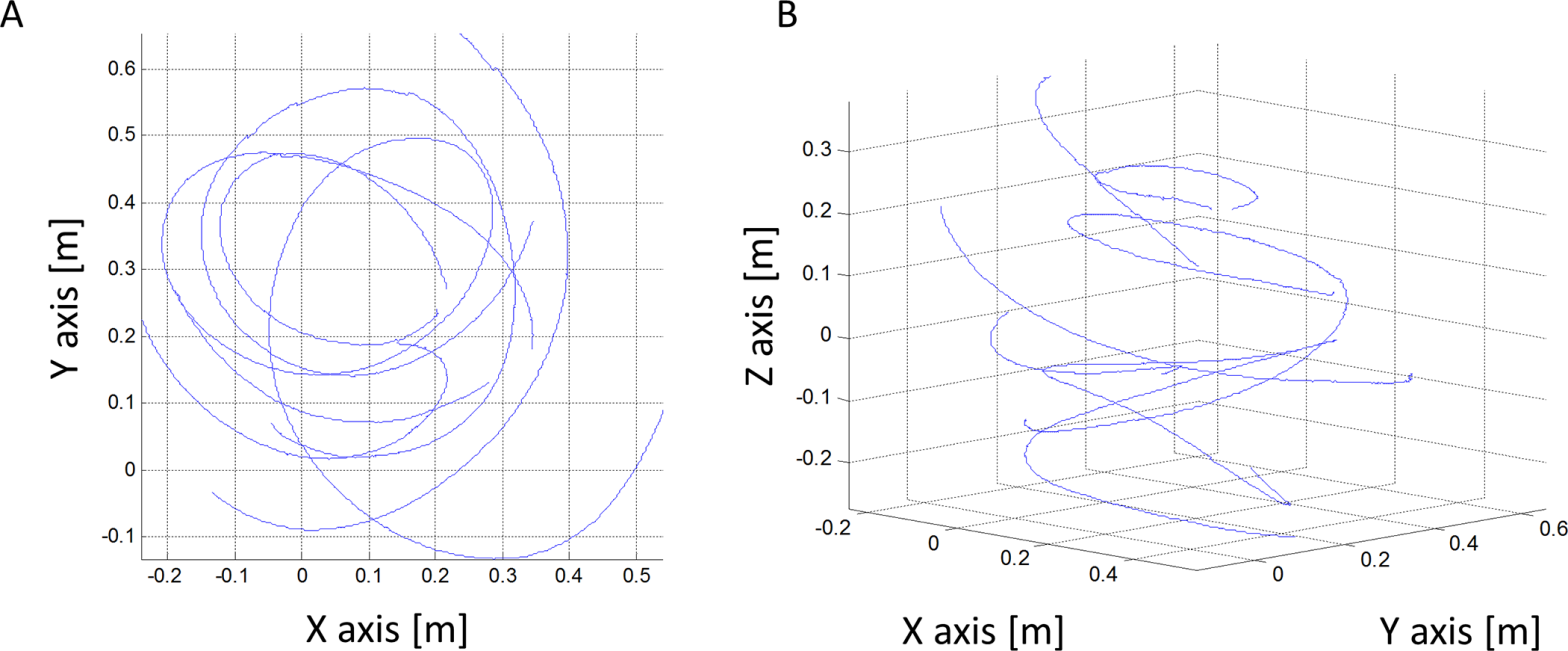
Free-flight maneuvers of females *R*. *ferrugineus* flying around a lamp in the laboratory. Blue lines are the flight trajectories tracked in 3-dimensions. A) The trajectories in the horizontal plane (i.e. viewed from above). B) The trajectories in 3D.

Fig 12 shows the free-flight trajectories of weevils flying around a lamp in a large room. While circulating the lamp the beetles made banked turns (S1 Video). The average turning radius of the trajectories was 0.49 ± 0.11 m (n = 13). During these maneuvers the beetle had an average flight speed of 1.54 ± 0.21 ms^-1^ and a mean centripetal acceleration of 5.6 ± 0.62 ms^-2^. If the beetles were to maneuver at constant flight speed without losing altitude they would need to bank the body by 30° and increase lift to 115% of the body weight so that this lift would provide both the vertical component needed to counter the weight and the horizontal force needed to provide the centripetal acceleration (Fig 1). However, most of the free flying beetles were losing altitude during the maneuver (Fig 12B). Five of the beetles were either gaining elevation or keeping their trajectory horizontal while maneuvering. The mean turning radius for these beetles was lower (0.24 ± 0.04 m, n = 5) and the mean flight speed was 0.9 ± 0.10 ms^-1^ so that the mean centripetal acceleration was only 3.7 ± 0.64 ms^-2^. This implies a banking angle of 21° and a lift force equivalent to 107% of the body weight, if the beetles are to circle horizontally without losing altitude.

## Discussion

Flight-mills present convenient tools by which to evaluate the flight behavior and flight performance of insects. However, the design of the flight-mill can affect the flight of the insect within the device. Our results show that the lift forces generated by the beetles were lower than the force needed to support their body weight in air. In contrast, the thrust needed to move the insect + flight-mill forward was higher than in free flight at the same flight speed. In addition, we measured distinct differences in flapping kinematics associated with the circular path and tethering orientation of the beetles. Interestingly, these differences in wingbeat kinematics did not result in significant differences in flight speed or wingbeat frequency. Hence, the adjustments made in wingbeat kinematics compensated for differences in flight-mill design to provide a similar flight output, as measured by the flight-mill. However, from this experiment, we do not know how the observed changes in wing-beat kinematics would have affected prolonged flight. The flight output achieved by different kinematic solutions may not be equally efficient, resulting in different energetic costs. These small differences in energetic costs can become substantial during prolonged flight, as evidenced by our mass loss experiment.

While most flight-mill studies acknowledge that flight-mill flight cannot be perceived as equivalent to free-flight, only a few studies have actually attempted to quantify the difference between the two. Riley et al. [14] and Chance [16] measured the power needed to keep the flight-mill rotating at a constant angular speed. Chance [16] used arguments provided by Hocking [28] to estimate the equivalent free-flight speed of *Agrotis orthogonia* from its flight-mill flight speed. He estimated free-flight speed to be 20% higher than flight-mill speed. The biomechanical argument, however, ignored the need to support body weight in the air and the effect of increased flight speed on the propulsive and aerobic efficiency [29,30] of the insects. Taylor et al. [15] compared flight-mill speeds of the emerald ash borer (*Agrilus planipennis*) with the measured speed of the same beetles during free-flight. The mean free-flight speed was ~3-fold higher than the mean flight-mill speed. The free-flight speeds were measured during short bursts (<3 seconds) of flight associated with take-off, whereas most of the insects in the flight-mills flew more than 750 m [15]. Therefore, it is unknown whether the measured free-flight speeds are indicative of flight at cruising speed. Riley et al. [14] compared the flight speed of *Cicadulina* leafhoppers in the flight-mill to published free-flight speeds of similarly-sized insects. The estimated free-flight speed was again 3-fold higher than the measured flight-mill speed. However, the calculations also showed that the power needed to overcome the drag of the body in the free-flight speed (1.3 μW) was 1.4-fold higher than that delivered to the flight-mill to keep it rotating at the flight-mill speed (0.9 μW). Furthermore, adding data from free-flight climb speeds indicated that supporting body weight in the air can elevate the power output of free-flight to a value 2.6-fold higher than that power needed to rotate the flight-mill. An insect in the flight-mill might of course also invest the power to counter its body weight, but there is no way of knowing if this is indeed the case. Thus, while free-flight speed is expected to be higher than flight-mill speed, there is no way of knowing whether the insect in the flight-mill expends the same energetic power as in free flight.

There are ample reports suggesting that tethered insects indeed fly at reduced effort compared to free-flight. The metabolic rate of tethered bumblebees was roughly half that of during free-flight [13]. Moths flying in a flight-mill had thoracic temperatures much lower than in free-flight [31]. Tethered locusts in a wind tunnel which flew at preferred flight speed, often produced lift forces that were as low as 50% of their body weight. Their thorax temperature and, therefore, metabolic rate were also much lower during these flights [32]. These reports support our finding that while more energy is invested by the insect to rotate the flight-mill, the overall cost of flight can be reduced or kept the same by lowering the level of lift produced. It is therefore important to evaluate the contribution of lift during flight-mill flight. Here, the vertical force measured directly from the balance of vertical forces of the flight-mill in the seesaw design showed that the lift produced was insufficient to counter the weight of the insect. Average vertical forces were found to be equivalent to 58% and 65% of body weight, for banked and level beetles, respectively. The value of vertical force for the banked beetles implies a mean lift of 76% of the body weight. Since the beetles were flying at similar flight speed, this suggests that the total power output of the banked beetles was higher than for level beetles for flight at the same speeds.

The other estimate of vertical force for the same flights (74% and 55% of body weight, for the level and banked flight respectively) was derived indirectly from the aerodynamics of the wing motion. Here too forces smaller than the body weight were found, but with no difference in lift between level and banked flight. Undeniably, there is a large margin of error in relating flapping kinematics to quasi-steady aerodynamic forces [see the proof by contradiction explained in [25,29]]. This is particularly true here, where models from hovering flight were used to infer forward flight and wing rotation and inertial effects were ignored. Nevertheless, the quasi-steady analysis is still informative as a comparative tool because it allows interpretation of the joint effect of changes in multiple kinematic parameters occurring concurrently. Furthermore, it allows to compare the aerodynamic output of the left and right wings allowing us to identify steering attempts.

The significant interactions between wing side and body orientation revealed that the asymmetry of flapping was more profound when the beetles were flying level (Figs 5–7). The quasi-steady force estimation suggests that these asymmetries primarily resulted in asymmetric thrust generated by the left and right wings with the wing internal to the curved flight path producing larger forward thrust and the external wing producing negative thrust (i.e. drag) in some cases (Fig 10B). While the estimated quasi-steady thrust forces were somewhat lower than the thrust needed to keep the flight-mill rotating (average for the estimated thrust 7–12.5% of the body weight in the different flight-mills), there were no significant differences in thrust between the fixed and seesaw condition (p = 0.11) or between banked and level orientations (p = 0.9). This agreed with our observation that flight speeds did not vary among the flight-mill variants.

The lateral asymmetries in thrust production were more pronounced when the beetles were level, leading to a yaw (rotation about the vertical axis) torque on the beetles to turn opposite to, and exit the curved flight trajectory (Fig 10B). One important conclusion from our study was thus that tethering the beetles in the banked turn orientation helped to mitigate the asymmetric flapping. This should not be surprising given that free-flying beetles were observed to perform bank turns during curved flight paths in the laboratory (S1 Video).

Due to the angular motion associated with the circular trajectory, the beetles experienced a lower forward flight speed at the left wing (internal to the circular trajectory) compared to the right wing. Thus, a beetle flapping in lateral symmetry would experience lower wingtip speeds relative to air in the left wing. The bilateral asymmetry in flapping in our beetles not only compensated for the lower flight speed on the left side, but actually resulted in higher speed relative to air in the left wing during the upstroke. During forward flight with a tilted stroke plane much of the thrust is generated during the upstroke while most of the lift is generated during the downstroke [33]. This again suggests that the beetles were actively attempting to steer (yaw) out of the circular trajectory.

As depicted in Fig 1 and in the pendulum analysis above, banked beetles should increase their lift force to reach the same up-thrust as in the level flight orientation. Thus, banked beetles do not just flap at higher symmetry; they should also invest more force in achieving a vertical force balance than level beetles. This higher force makes their flight more relevant to free-flight conditions and results in a higher energetic price compared to level-tethered flight. Indeed, banked beetle lost more mass during prolonged flight (Fig 11). While banking seems to reduce flapping asymmetry this does not imply that the circular flight trajectories imposed by flight-mills are equivalent to free-flight maneuvers. For a free flying beetle flying in a circle with a radius = 0.4 m and flight speed of 1.85 ms^-1^ the centripetal acceleration is 8.56 ms^-2^. To provide this acceleration while keeping the flight speed constant, and without losing altitude, the weevil must generate lift that is equivalent to 133% of the body weight and the body should be banked by 41°. While the free flying beetles maneuvered at comparable and even smaller turning radii (Fig 12) they also lost height or slowed down so that the centripetal acceleration during levels flight was on average 3.7 ms^-2^. Even at these lower flight speeds the lift is expected to increase to 1.07 of the body weight. In contrast, both the direct measurements from the flight-mill dynamics and the estimates from the wingbeat kinematics show that the lift generated by the beetles in the flight-mill was much lower than 100% of the body weight. Thus the flight-mill mechanism, provides both the vertical and horizontal support that allows the beetles to make more demanding maneuver for less aerodynamic force.

While flight-mill flight in a banked body orientation may be more appropriate to mimic symmetric free-flight conditions it does not solve the fact that flights occur at lower speeds compared to straight free-flight due to the added resistance of the flight-mill. Typically, thrust comprises only a small portion of the total flight force in insects. In locusts, estimates as well as indirect measurements of thrust suggest that thrust at cruising flight speeds constitutes only 7% of the lift force [33]. The force needed to keep the flight-mill rotating at the constant flight speed in our experiment was more than double that value. Nevertheless, the resultant aerodynamic force (trust and lift combined) was still less than 100% of the body weight implying that the beetles in the flight mill are flying using aerodynamic forces that would be insufficient to keep their body in the air during free-flight at the same speed. The flight-mill speeds measured here were somewhat lower than flight speeds measured on a much larger sample size (N = 56) using a similar flight-mill (2.15 ms^-1^; Barkan et al. in review). They were also similar and 83% higher than two previous studies on female red palm weevils flying in different flight-mills (1.79 ms^-1^ in the summer [3] and 1.01 ms^-1^ [1]). Hence, the resistance of our flight-mill is not higher than that of flight-mills used in the past to study the same insects. We thus believe that our findings on altered flapping kinematics as a result of flight-mill design are also applicable to other flight-mill studies.

## Conclusion

We found that lift production was below the expected values for free-flight and that the beetles steered in the opposite direction of the curved flight path by flapping asymmetrically. Both the flapping asymmetry and low lift can be somewhat rectified by tethering the beetle in a banked orientation. The latter results in a higher energy expenditure per distance and time flown, but still does not correspond directly to free-flight because the force needed to rotate the flight-mill is higher than air resistance at the same flight speed during free-flight. However, the flight-mill resistance is relatively simple to measure and should provide a simple way to correct for the effect of different flight-mills on the flight performance of the insects measured in them. Flight-mills will continue to be an important tool in assessing the flight physiology, and potential of insects to fly, but their limitations as described here should be taken into careful consideration when designing the flight-mill and when attempting to infer from flight-mill studies the dispersal ranges of insects in situ.

### Appendix A–flapping kinematics

The wing-flapping kinematics can be described as three time-varying angles of the wing about the wing base. To extract these angles we first transformed the three digitized points on the wing, found in the lab-based coordinate system (X,Y,Z), to a body frame of reference with the origin at the wing base. To do so, we first shifted the lab-based coordinate system to a point between the two wing bases in each video frame. We then used the 3D positions of the left (B_L_) and right (B_R_) wing bases to determine the instantaneous unit vector that represents the lateral axis of the beetle Y_B_. The direction of the longitudinal (X_B_) and the dorso-ventral axes (Z_B_) of the body were found as in Ellington [21] from the cross-product of

$$X_{B}=Y_{B}\times Z$$ (6)

where *Z* is the unit vector of the vertical (in the lab-based coordinate, i.e. *Z* = [0, 0, 1]) the dorso-ventral axis (*Z*_*B*_) was defined as:

$$Z_{B}=X_{B}\times Y_{B}$$ (7)

The directional cosine matrix of the body axes was used to rotate all the positions of points on the wings to the insect’s frame of reference (X_B_, Y_B_, Z_B_):

$$P_{B}=\left[\begin{array}{ccc} {b1}_{x} & {b1}_{y} & {b1}_{z}\\ {b2}_{x} & {b2}_{y} & {b2}_{z}\\ {b3}_{x} & {b3}_{y} & {b3}_{z} \end{array}\right]\left[\begin{array}{c} P_{X}\\ P_{Y}\\ P_{Z} \end{array}\right]$$ (8)

where *P* is any 3D position in the lab frame of reference (X,Y,Z) fixed to the body, and *P*_*B*_ is its new (transformed) 3D position in the body frame of reference. The rotation matrix is composed of the directional cosines of the three body axes, i.e.: *X*_*B*_ = [*b*1_*x*_,*b*1_*y*_,*b*1_*z*_], *Y*_*B*_ = [*b*2_*x*_,*b*2_*y*_,*b*2_*z*_] and *Z*_*B*_ = [*b*3_*x*_,*b*3_*y*_,*b*3_*z*_].

Next, we shifted the data points of each wing to have an origin at the wing base (B_L_ and B_R_). We then found the stroke plane angle (β, see Fig 3A) of the left and right wings from the least square regressions of the positions of point T_L_ and T_R_ in the X_B_Z_B_ plane.

Data points from the two wings were rotated to the stroke plane, e.g.:

$$T_{s}=\left[\begin{array}{ccc} \cos\beta & 0 & \sin\beta \\ 0 & 1 & 0\\ -\sin\beta & 0 & \cos\beta \end{array}\right]\left[\begin{array}{c} T_{XB}\\ T_{YB}\\ T_{ZB} \end{array}\right]$$ (9)

where point *T*_*s*_ is the wing tip (*T*) rotated from the body axes (X_B_,Y_B_,Z_B_) by the stroke plane angle. Similarly, we rotated point C on the wing’s trailing edge.

The instantaneous flapping angle in each frame (*φ*, Fig 3A) was defined as in as in Ellington [21] and Fry et al.[22,23]: i.e., from the projection of the line connecting the wing base and the wing tip onto the stroke plane. Similarly, the angular deviation of this line forming the stroke plane was defined as the deviation angle (*θ*), and the geometric angle of incidence was found from the wing chord connecting point C and a point half-way on the leading edge between the wing base and wing tip. The wing was rotated by the instantaneous flapping angle as in Walker et al. [34] and the angle of incidence was found from the angle of the wing chord with the stroke plane (Fig 3A).

Wing frequency was found from the number of video frames in a flapping cycle. The ventral and dorsal stroke reversal points were found from the instantaneous flapping angles during the stroke reversal points. The flapping amplitude was defined as the angular displacement between the ventral and dorsal stroke reversal points (Fig 3B). To compare the angle of incidence between different flights we used the angle of incidence at mid-stroke during the upstroke and downstroke.

### Appendix B–conical pendulum calculations

A simple conical pendulum is a mass suspended by a massless string from a higher pivot point. If the mass moves in a horizontal circle at constant speed the tension in the string provides a horizontal centripetal force and a vertical force equal to the weight of the mass. These two forces determine the angle of the string with the vertical (*γ*).

The weight of the mass is:

W=mg (10)

The centripetal force (*F*_*c*_) can be written in the form:

$$F_{c}=m\omega^{2}l\;\sin\gamma$$ (11)

where *m* is mass, *ω* is the angular speed of the flight-mill, *l* is the length of the arm to which the mass is tethered and, therefore, *l* sin *γ* is the turning radius of the mass.

If the mass is a beetle flapping its wings it may produce some aerodynamic up-thrust (lift), this shifts the balance of forces in the vertical plane, resulting in a different (larger) *γ* angle. Thus, the amount of vertical up-thrust imparted by the beetle during the flight (*F*_*Va*_) can be solved from the mass of the beetle, the rotation speed of the flight-mill, and the observed angle *γ*. This is achieved by balancing the torques about the pivot point in the flight-mill with the seesaw design. Note, that the flight-mill is balanced prior to attaching the beetle so that the gravitational moments of both sides of the flight-mill arm are balanced and therefore cancel one another out.

$$m_{1}gl_{1}\;\sin\gamma -m_{2}gl_{2}\;\sin\gamma =0$$ (12)

Hence, we are left with the gravitational torque due to the weight of the beetle with mass *m*_3_ at the end of the radial arm (at distance *l*_3_) from the pivot. However, the torques due to centrifugal force are not cancelled out, therefore

$$m_{1}\omega^{2}l_{1}^{2}\;\sin\gamma \;\cos\gamma +m_{2}\omega^{2}l_{2}^{2}\;\sin\gamma \;\cos\gamma +m_{3}\omega^{2}l_{3}^{2}\;\sin\gamma \;\cos(\gamma -\vartheta )-m_{3}gl_{3}\;\sin(\gamma -\vartheta )+F_{Va}l_{3}\;\sin\left(\gamma -\vartheta \right)=0$$ (13)

where *F*_*Va*_ is the aerodynamic up-thrust imparted by the beetle and the angle *ϑ* corrects for the fact the beetle being tethered slightly below the tip of the radial arm and therefore *l*_3_ having a slightly smaller angle with the vertical.

After rearranging and isolating *F*_*Va*_:

$$F_{Va}=-\frac{\omega^{2}\left[\sin\gamma \;\cos\gamma \left(l_{2}^{2}m_{2}+l_{1}^{2}m_{1}\right)+l_{3}^{2}m_{3}\sin\left(\gamma -\vartheta \right)\cos\left(\gamma -\vartheta \right)\right]-m_{3}gl_{3}\sin\left(\gamma -\vartheta \right)}{l_{3}\sin\left(\gamma -\vartheta \right)}$$ (14)

The angle *ϑ* equals 6.5° and 4.5° when the beetles are in the level and banked orientation respectively., *l*_1_, *l*_2_, and *l*_3_ are the distance from the pivot to the center of mass of *m*_1_, *m*_2_ and *m*_3_, which are the mass of the radial arm, the mass of the counter arm (with weights), and of the beetle, respectively (Table 3).

**Table 3:** Dimensions and mass of parts of the flight-mill used in the study. The data is substituted in Eq 14 to find the vertical force generated by the flying beetle.

	Level	Banked
*l*_1_ [cm]	28.4	28.4
*l*_2_ [cm]	7.5	7.1
*l*_3_ [cm]	40	40
*m*_1_ [g]	6.68	6.4
*m*_2_ [g]	25.26	25.55
*m*_3_ [g]	Beetle’s body mass	Beetle’s body mass

### Appendix C–Quasi-steady force estimation

To convert the effect of change in flapping kinematics into meaningful insight on aerodynamic force production, we estimated the quasi-steady aerodynamic forces associated with the translation of the wing through air. This was done by determining the instantaneous flapping velocity of the wing at the second moment of wing area and adding the forward velocity of the body to give the speed of the wing relative to air. The instantaneous angle of attack was found between this relative velocity vector and the wing chord at point C (Fig 3). The forward speed of the body was added to the left and right wings, based on the angular speed of the flight-mill and distance from the pivot point, to account for differences in tangential speeds between the left (internal) and right wing due to the circular trajectory.

The quasi-steady aerodynamic force due to wing translation can be estimated for each wing in each video frame from Eqs 4 and 5. However, the lift and drag coefficients at different angles-of-attack are unknown. Since we were only interested in comparing the aerodynamic output of the wings we used a generalized trigonometric function. Sane [25] reviewed published data on the change in lift and drag coefficients with the angle of attack in several insects (see his Fig 9). Those data in his figure specific for flapping wings suggest that the relationship between the force coefficients and the angle of attack can be roughly described as

$$C_{L}=k_{1}\sin\alpha \;\cos\alpha$$ (15)

and

$$C_{D}=k_{2}\sin^{2}\alpha +k_{3}$$ (16)

where *C*_*L*_ and *C*_*D*_ are the lift and drag coefficients and *α* is the angle of attack of the wing. The constant *k*_1_ sets the maximal lift coefficient, and *k*_2_ and *k*_3_ set the maximum and minimum of the drag coefficient in the curves.

The values vary between insects and experiments:

*k*_1_ varies between 2.6 and 3.6, we used 3.0.

*k*_2_ changes between 3.2 and 3.5, we used 3.35

*k*_3_ changes between -0.2 and 0.4, we used 0.1

The resultant of the lift and drag force is taken to be perpendicular to the wing surface [25,35] The vertical and horizontal component of this resultant force is taken to be the lift and thrust of the beetles, respectively.

## Supporting information

S1 Video Supporting video. High-speed movie showing free flying red palm weevils making banked turns while circulating a lamp.
(MP4)

S1 Data Raw data. Data derived from the flight-mill and flapping kinematics. These data are used to derive the conclusions of the study.
(XLSX)
